# The oral cavity as a reservoir for resistance- and hypervirulence-associated genes of *Klebsiella pneumoniae* in hospitalized patients

**DOI:** 10.3389/fmicb.2026.1751947

**Published:** 2026-02-05

**Authors:** Karima Zenati, Sascha D. Braun, Djellali Belhadi, Amira A. Moawad, Elke Müller, Celia Diezel, Christian Brandt, Raouya Mostefaoui, Stefan Monecke, Fatma Zohra Zaidi, Mohamed Belmahdi, Abdelaziz Touati, Ralf Ehricht

**Affiliations:** 1 Laboratory of Microbial Ecology, Department of Microbiology, Faculty of Natural and Life Sciences, University of Bejaia, Bejaia, Algeria; 2 Leibniz Institute of Photonic Technology, Member of the Research Alliance “Leibniz Health Technologies” and the Leibniz Centre for Photonics in Infection Research (LPI), Jena, Germany; 3 Center for Translational Medicine (CETRAMED), Jena University Hospital, Friedrich Schiller University Jena, Jena, Germany; 4 InfectoGnostics Research Campus Jena e.V., Jena, Germany; 5 Friedrich-Loeffler-Institut, Institute of Bacterial Infections and Zoonoses, Jena, Germany; 6 Animal Health Research Institute, Agriculture Research Center (ARC), Giza, Egypt; 7 Institute of Infectious Diseases and Infection Control, Jena University Hospital, Friedrich Schiller University, Jena, Germany; 8 Institute for Medical Microbiology and Virology, Dresden University Hospital, Dresden, Germany; 9 Department of Biology, Faculty of Natural and Life Sciences, University of Djelfa, Djelfa, Algeria; 10 Institute of Physical Chemistry, Friedrich Schiller University Jena, Jena, Germany

**Keywords:** blaNDM-5, blaOXA-48, carbapenemases, hospital, hvKp, *Klebsiella pneumoniae*, oral cavity, saliva

## Abstract

**Introduction:**

This study investigated the epidemiology and distribution of carbapenem resistance and virulence genes in *Klebsiella pneumoniae* strains isolated from the oral cavity of hospitalized patients, highlighting their role as reservoirs in non-epidemic contexts.

**Methods:**

Carbapenem-resistant *Klebsiella* spp. were isolated from the oral cavity of 180 hospitalized patients in medical wards at two hospitals in Bejaia, Algeria. Screening for carbapenem resistance was performed on oral mucosa and saliva using Carba-MTL broth. Antibiotic susceptibility was assessed with the Vitek2 system and interpreted according to EUCAST guidelines. Whole genome sequencing (WGS) was carried out using Oxford Nanopore Technologies, with ABRicate used for resistance/virulence gene detection and Kleborate for hypervirulence assessment. Whole-genome sequences were further examined to identify single-nucleotide polymorphisms (SNPs) and to reconstruct a SNP-based phylogenetic tree in order to assess the genetic relatedness among the isolates.

**Results:**

Twenty *Klebsiella* strains were identified as *K. pneumoniae*. Among these, 85% were carbapenem-resistant, carrying *OXA-48* (80%) or *NDM-5* (5%), and all harbored *blaCTX-M-15*. WGS of the 20 *K. pneumoniae* strains revealed a broad resistome, including *β*-lactamases (*CTX-M-15, CMY-4, OXA-1, TEM-1*), sulfonamide (*sul1, sul2*), aminoglycoside (*aac(3)-IIa, aadA2, aph(3′)-VI, armA, strA, strB*), trimethoprim (*dfrA12, dfrA5, dfrA14*), and tetracycline (*tetA*). Quinolone resistance was linked to QRDR mutations (*gyrA S83I, parC S80I*) and plasmid-mediated genes (*qnrS1, qnrB10, qnrS10, aac(6′)-Ib-cr*). Five distinct sequence types (STs) were identified, including high-risk clones ST13 and ST48. Virulence profiling revealed *yersiniabactin* (85%), frequently linked to ICEKp elements (ICEKp4, ICEKp10), and colibactin (40%) among OXA-48 isolates. Notably, a single *K. pneumoniae* isolate harboring NDM-5 (K21) carried both hypervirulence markers (*ybt9/ICEKp3, iuc1, rmp1/kpvp-1*) and carbapenem resistance, documenting, for the first time in Algeria, the convergence of these traits in oral isolates. ICEKp was identified as the key vehicle for dissemination of *yersiniabactin* and *colibactin*, and a novel association between ICEKp and *kpvp-1* was observed. Capsular typing showed predominance of K57-O1/O2v2 among OXA-48 producers and K27/O4 among NDM-5 strains.

**Conclusion:**

This study provides the first evidence in Algeria of OXA-48- and NDM-5-producing *K. pneumoniae* in the oral cavity of hospitalized patients. The coexistence of carbapenem resistance and hypervirulence underscores the oral cavity as a critical reservoir, potentially fueling nosocomial infections and the dissemination of high-risk clones within hospitals and the wider community.

## Introduction

The human oral cavity is a complex and dynamic microbial ecosystem, encompassing distinct niches such as the buccal mucosa, tongue, and palate, which collectively support a dense and diverse microbiota (Lu and Xuan, 2019). While predominantly inhabited by commensals, shifts in microbial balance, particularly under hospitalization or immunocompromised conditions, can facilitate colonization by clinically significant Gram-negative bacilli, including *Klebsiella (K.) pneumoniae* (Zaatout, 2021; Katkowska et al., 2023).

*Klebsiella* species are diverse, with relatively large genomes that confer metabolic flexibility, biochemical diversity, and the ability to colonize clinical and environmental niches such as soil, plants, surface water, and medical devices (Choby et al., 2020; Mendes et al., 2023). In humans, these bacteria are typically found within the microbiota of healthy individuals and inconspicuously inhabit the digestive systems (Dong et al., 2022; Muraya et al., 2022; Li et al., 2023).

Recent studies have frequently reported the presence of *K. pneumoniae* in oral samples (Le et al., 2020; Wei et al., 2021; Haruta et al., 2023), although its role as a true member of the oral flora remains under debate (Tsuzukibashi et al., 2023; Liu et al., 2024). This organism is known to colonize the oral cavity in specific contexts, particularly in hospitalized patients or those with poor hygiene or reduced salivary flow, thereby creating opportunities for nosocomial transmission via saliva or oropharyngeal secretions (Amaral et al., 2009; Needleman et al., 2013; Altayar et al., 2020).

Currently, most research has focused on *K. pneumoniae*, which accounts for approximately 95% of *Klebsiella* infections and is recognized as the most common human pathogen within this genus (Effah et al., 2020; Wyres et al., 2020; Shah et al., 2025).

This opportunistic pathogen represents a major global health threat due to the rise of hospital-acquired infections associated with multidrug resistance, particularly in intensive care units (ICUs), as well as community-acquired infections linked to its high virulence (Wei et al., 2021; Zhu et al., 2024; Shah et al., 2025). A substantial percentage of *K. pneumoniae* infections occur in newborns, the elderly, and those with compromised immune systems and can cause a range of infections, including pneumonia, soft tissue and surgical wound infections, urinary tract infections, bloodstream infections, pyogenic liver abscesses, and sepsis (Muraya et al., 2022; Li et al., 2023; Spadar et al., 2023; Zhu et al., 2024).

A significant proportion of infections caused by *Klebsiella* species can be attributed to two primary pathotypes: Multidrug-resistant (MDR) and hypervirulent (hv) clones (Dong et al., 2022; García-Cobos et al., 2025). Carbapenemase-producing *Enterobacterales* pose a serious threat to global health, creating urgent challenges in clinical settings. Among these, *Klebsiella pneumoniae* carbapenemase (KPC), New Delhi metallo-*β*-lactamase (NDM), and oxacillinase-48 (OXA-48)-like enzymes are the most critical carbapenemases in terms of their spread and clinical impact (Alvisi et al., 2025; Ragueh et al., 2025). Typically, carbapenemases are encoded on mobile genetic elements, such as plasmids, which can be readily transferred between species through horizontal gene transfer (Xie et al., 2025). The World Health Organization (WHO) has listed *K. pneumoniae* producing carbapenemase and extended-spectrum beta-lactamases (ESBL) among the “priority pathogens” given their multidrug-resistant phenotype (World Health Organization, 2024). The prevalence of antimicrobial resistance (AMR) genes carried on plasmids and mobile genetic elements has earned *K. pneumoniae* its reputation as a “key trafficker” of AMR genes between *Klebsiella* species and other *Enterobacterales*, highlighting its significance in the spread and development of AMR (Muraya et al., 2022).

In contrast to multidrug-resistant *K. pneumoniae*, hypervirulent *K. pneumoniae* (hvKp) has emerged as a significant pathogen primarily associated with severe and invasive community-acquired infections in healthy individuals, such as endophthalmitis and liver abscesses (Hussain et al., 2023; Spadar et al., 2023). They have also been increasingly reported in hospital-acquired infections. These isolates are generally sensitive to most antibiotics but exhibit high virulence (Russo et al., 2024). The virulence factors include the capsule (K1, K2, K20 capsular types), lipopolysaccharide (LPS) with regulatory genes *rmp*A and *rmp*A2, and siderophores, predominantly aerobactin (Effah et al., 2020; Spadar et al., 2023).

In recent years, hypervirulent *K. pneumoniae* (hvKp) strains have evolved through the acquisition of carbapenem resistance, representing a concerning convergence that poses a significant public health threat (Douradinha, 2023; Hussain et al., 2023; García-Cobos et al., 2025). Multiple evolutionary pathways contribute to the emergence of these strains, primarily through the acquisition of resistance plasmids by hvKp, resulting in carbapenem-resistant hypervirulent *K. pneumoniae* (CR-hvKp), the acquisition of virulence plasmids by carbapenem-resistant *K. pneumoniae,* resulting in hypervirulent carbapenem-resistant *K. pneumoniae* (hv-CRKp), or the acquisition of hybrid plasmids carrying both virulence and resistance genes (Arcari and Carattoli, 2023; Mendes et al., 2023; García-Cobos et al., 2025). These strains not only combine hypervirulence and multidrug resistance but also exhibit high transmission rates, making them a serious public health concern (Hussain et al., 2023). Although hvKp and carbapenem-resistant *K. pneumoniae* (CRKp) populations are recognized for their distinct clonal groups and sequence types (STs), primarily circulating in community and hospital settings respectively, virulence- and resistance-associated genes can be horizontally transferred and exchanged between these populations (Dong et al., 2022; Kochan et al., 2023; Spadar et al., 2023). Genomic analysis of *K. pneumoniae* strains reveals that these bacteria are classified into sequence types that form high-risk clones globally: the multidrug-resistant (MDR) clones (ST258, ST147, ST101) predominantly circulating in the Western world and hypervirulent clones (ST23, ST65, ST86) mainly in Eastern regions (Arcari and Carattoli, 2023; Byarugaba et al., 2023). The most common MDR-hvKP strains worldwide include the KPC-producing ST11 strain. However, due to the dissemination of mobile genetic elements encoding NDM-1 and OXA-48-type carbapenemases, hvKP strains harboring these determinants have increasingly emerged in recent years (Mendes et al., 2023; García-Cobos et al., 2025). These strains have been reported in several African countries, including Algeria (Zemmour et al., 2021), Egypt (Ahmed et al., 2021; Mendes et al., 2023), Kenya, and Sudan (Ofosu-Appiah et al., 2024).

In Algeria, OXA-48 represents the most common carbapenemase type circulating (Touati and Mairi, 2020). Several studies have reported the isolation of OXA-48-producing *K. pneumoniae* from clinical samples (Agabou et al., 2014; Bouguenoun et al., 2016; Loucif et al., 2016; Yousfi et al., 2019; Abderrahim et al., 2022), the hospital environment (Bouguenoun et al., 2016), and different ecological niches (Mairi et al., 2019; Loucif et al., 2022a).

Our study aimed to investigate the presence, genetic diversity, resistance mechanisms, and virulence traits of *K. pneumoniae* colonizing the oral cavity of hospitalized patients in Algeria, with a specific focus on the emergence of high-risk clones exhibiting convergence of multidrug resistance and hypervirulence.

## Materials and methods

### Patients and clinical specimens

The study was conducted in two public hospitals located in Kherrata and Akbou, in Bejaia province, Algeria. This investigation was part of an ongoing prospective surveillance of nosocomial infections and the spread of multidrug-resistant bacteria within the hospital setting. The study was conducted for ecological purposes and involved only anonymized, non-pathological biological samples. Between 2023 and 2024, a total of 180 hospitalized patients in the medical ward were sampled. Two samples were collected from the oral cavity, including saliva (*n* = 69) and oral mucosa swabs (*n* = 111) from the healthy buccal cavity of these patients. The patients included both genders (male and female) and ranged in age from 18 to 89 years. Clinical information was collected, including hospitalization ward, antibiotic use, age, and gender. However, detailed information regarding the reason for hospitalization was available for only a limited subset of patients.

The participants were instructed to abstain from oral hygiene for 24 h and to refrain from eating, drinking, or rinsing their mouths for 1 h prior to examination. To collect the samples, unstimulated saliva was obtained by passive drooling into 15 mL sterile conical tubes, while sterile cotton swabs were used to sample different areas of the oral mucosa, including the buccal mucosa, tongue, palate, and oral vestibule. All samples were immediately transported at 4 °C to the Microbial Ecology Laboratory at the University of Bejaia for analysis.

### Bacterial isolates and culture conditions

Mucosal swabs and saliva were cultured directly in 2 mL of nutrient broth (Fluka, St Louis, USA) and incubated for 24 to 48 h at 37 °C. After that, 50 μL of the pre-enrichment broths were collected and added to 1 mL of Carba-MTL broth (Mairi, Touati, Lavigne) and then incubated at 37 °C for 12 h for preliminary screening of carbapenemase-producing *Enterobacterales* (Mairi et al., 2018). Positive Carba-MTL broths were then subcultured on MacConkey agar (Fluka, St Louis, USA) supplemented with 0.5 mg/L of ertapenem (bioMérieux) and 32 mg/L of vancomycin (bioMérieux). The plates were incubated at 37 °C for 18–24 h, after which colony growth was observed. In cases where mixed cultures were observed, subclones were isolated and analyzed separately to ensure accurate strain-level identification and characterization.

The resulting colonies were then identified firstly by screening on Chromagar TM orientation, associated with other conventional biochemical tests, and confirmed by MALDI-TOF MS Ultraflex instrument (BrukerDaltonics GmbH, Bremen, Germany).

### Phenotypic antimicrobial susceptibility testing

Antimicrobial susceptibility testing was performed by agar disk diffusion according to the European Committee on Antimicrobial Susceptibility Testing (EUCAST, 2024), and minimum inhibitory concentration (MIC) testing of isolates was performed using a VITEK-2 system (bioMerieux, Nürtingen, Germany). The tested antimicrobial agents included in the VITEK 2 card AST-N430 were ampicillin, ampicillin/sulbactam, piperacillin/tazobactam, imipenem, gentamicin, cefuroxime, ciprofloxacin, cefuroxim-axetil, cefpodoxime, cefotaxime, ceftazidime, tigecycline, fosfomycin, nitrofurantoin, and trimethoprim/sulfamethoxazol. Interpretation of results was carried out according to the EUCAST standard 2024.

Phenotypic detection of extended-spectrum *β*-lactamase (ESBL) and carbapenemase was carried out using the synergy test (Jarlier et al., 1988) and the carbapenem inactivation method (CIM) (Van Der Zwaluw et al., 2015), respectively.

### Sequencing

Whole-genome sequencing of all strains listed in Supplementary Table 1 was performed using the Oxford Nanopore Technologies (ONT) MinION platform to confirm species identity and characterize resistance gene profiles. Genomic DNA was extracted using the NucleoSpin Microbial DNA Kit (Macherey-Nagel, Düren, Germany) with minor protocol modifications. Bacterial isolates were cultured overnight on Columbia blood agar (Becton Dickinson, Heidelberg, Germany), and biomass was collected using a full inoculation loop. Cells were suspended in 500 μL PBS (pH 7.4), pelleted by centrifugation, and resuspended in 100 μL buffer BE. Mechanical lysis was achieved using a BeatBeater (Biozym, Hessisch Oldendorf, Germany) for 5 min at maximum speed. Proteinase K digestion was followed by heat inactivation at 70 °C for 5 min. RNase A (100 mg/mL; Sigma-Aldrich, Steinheim, Germany) was then added and incubated at 37 °C for 5 min. DNA was purified and eluted in 70 μL of nuclease-free water (Carl Roth, Karlsruhe, Germany).

Library preparation was conducted using the SQK-NBD114.24 Native Barcoding Kit (Oxford Nanopore Technologies), and sequencing was performed exclusively on R10.4.1 flow cells (FLO-MIN114). DNA was size-selected using AMPure XP beads (Beckman Coulter, Krefeld, Germany) at a 1:1 ratio to enrich for high-molecular weight fragments. Sequencing runs were executed for 72 h using MinKNOW (v23), and raw signal data were recorded in POD5 format.

### Bioinformatics

Basecalling was performed using Dorado v0.9.5 (Oxford Nanopore Technologies) with the high-accuracy basecalling model res_dna_r10.4.1_e8.2_400bps_sup@2023-09-22_bacterial-methylation. Initial quality control of basecalled reads was carried out using FastQC v0.12.1 and NanoComp v1.12.0 to evaluate per-base quality scores, read length distributions, and barcode performance across samples. *De novo* genome assemblies were constructed using Flye v2.9.1-b1780. To enhance accuracy, the assemblies were subjected to four iterative rounds of polishing using Racon v1.5.0 with optimized parameters (match = 8, mismatch = 6, gap = 8, window = 500) to correct long-read-specific basecalling errors. Final polishing was completed using Medaka v1.8.2, applying the model r1041_e82_400bps_bacterial_methylation, which corrects residual errors, including indels and methylation artifacts. The resulting high-quality assemblies were used for downstream genomic analyses.

Annotation of resistance and virulence genes was performed using Abricate v1.0.1 against multiple curated databases, including NCBI AMRFinderPlus (2024-05-01 release), CARD (v3.2.9), ResFinder (v4.2, 2024 release), and the Virulence Factor Database (VFDB) (2024-03-01). Default thresholds were applied, requiring a minimum of 90% sequence identity and 70% coverage to report gene presence.

Multilocus sequence typing (MLST), capsular (K) and O-locus determination, as well as virulence and resistance scoring were conducted using Kleborate v2.3.2 (Lam et al., 2021), which integrates genomic profiling with scoring algorithms to categorize isolates based on clinically relevant traits. Virulence scores (ranging from 0 to 5) were derived from the presence of siderophore systems such as yersiniabactin (*ybt*), colibactin (*clb*), aerobactin (*iuc*), and salmochelin (*iro*), as well as hypermucoidy genes (*rmp*ADC), while resistance scores (ranging from 0 to 3) were assigned based on the detection of ESBLs and carbapenemase genes. Capsular and lipopolysaccharide antigen loci were identified using the integrated Kaptive module (v0.7.3), enabling the assignment of specific KL and O-types across the dataset. The presence of mobile genetic elements such as integrative conjugative elements (ICEKp) and virulence plasmids (KpVP-1) was also assessed based on chromosomal context and alignment to reference sequences in the Pasteur Institute’s Klebsiella genomic repository.

### Whole-genome SNP-based phylogenetic analysis

Whole-genome sequences were examined to identify single-nucleotide polymorphisms (SNPs) and to reconstruct a phylogenetic tree. Initial clustering of genomes was performed using k-mer similarity with Sourmash (v4.8.11). Core genome SNPs, defined as regions conserved across all isolates, were aligned with Snippy-core (v4.4.1; github.com/tseemann/snippy). Phylogenetic analyses were based on fully assembled genomes, with SNP calling performed against a *de novo* assembled Reference Isolate (ID 99029) from within the study cohort to maximize mapping quality and core genome resolution.

A maximum-likelihood phylogeny was then inferred from this core SNP alignment using FastTree (v2.1.10) under the generalized time-reversible (GTR) nucleotide substitution model (Price et al., 2009). The resulting tree was visualized in Microreact (Argimón et al., 2016) (https://microreact.org/project/kThGFhfzumxwmtfCdDXzEm-klebsiella-pneumonia-algeria, last updated September 25, 2025) to enable interactive data exploration.

## Results

### Epidemiological investigation

Medical information, fully anonymized prior to analysis, was obtained from hospitalized patients, including gender, age, antibiotic therapy, and the origin of their hospitalization (Table 1). The study included 98 female (54.4%) and 82 male (45.5%) patients aged between 18 and 89 years, with the most common reasons for hospitalization among positive patients harboring *K. pneumoniae* being diabetes mellitus and thyroid disorders (Supplementary Table 1).

**Table 1:** Demographic and clinical characteristics.

Characteristic	Clinical Data
Age (range)	18–89 years
Gender	
Female	98 (54.44%)
Male	82 (45.55%)
Department	
Medical ward	180 (100%)
Source of isolate	
Saliva	69 (38.33%)
Oral mucosa swab	111 (61.66%)
Hospital source	
Akbou hospital	42 (23.33%)
Kherrata hospital	56 (31.11%)
Anti-biotherapy during the hospitalization	
Yes	1 (0.56%)
No	179 (99.44%)
Patients carrying CRKP (17 isolates)	
Hypertension	3 (17.64%)
Diabetes mellitus	5 (29.41%)
Thyroid disorders	4 (23.53%)
Respiratory diseases	1 (5.88%)
Chronic heart failure	2 (11.76%)
Surgery	1 (5.88%)
Scleroderma	1 (5.88%)
Stroke	1 (17.64%)

### Clinical characteristics of the oral *Klebsiella* spp.

Twenty *Klebsiella* sp. strains were isolated from 180 hospitalized patients in the medical wards. They were characterized using MALDI-TOF-MS and identified based on Kleborate results derived from whole genome sequencing (WGS) data as *K. pneumoniae*.

Some participants had multiple strains isolated from a single sample. The distribution of isolates included eight participants with a single isolate, five with two isolates, and one with three isolates (Supplementary Table 1). We isolated a total of 14 *K. pneumoniae* from oral mucosa swabs and six from saliva samples. Additionally, we observed that two patients had two isolates from the same type of sample.

### Antimicrobial resistance patterns (AMR) and resistome of *Klebsiella* isolates

**Figure 1:**
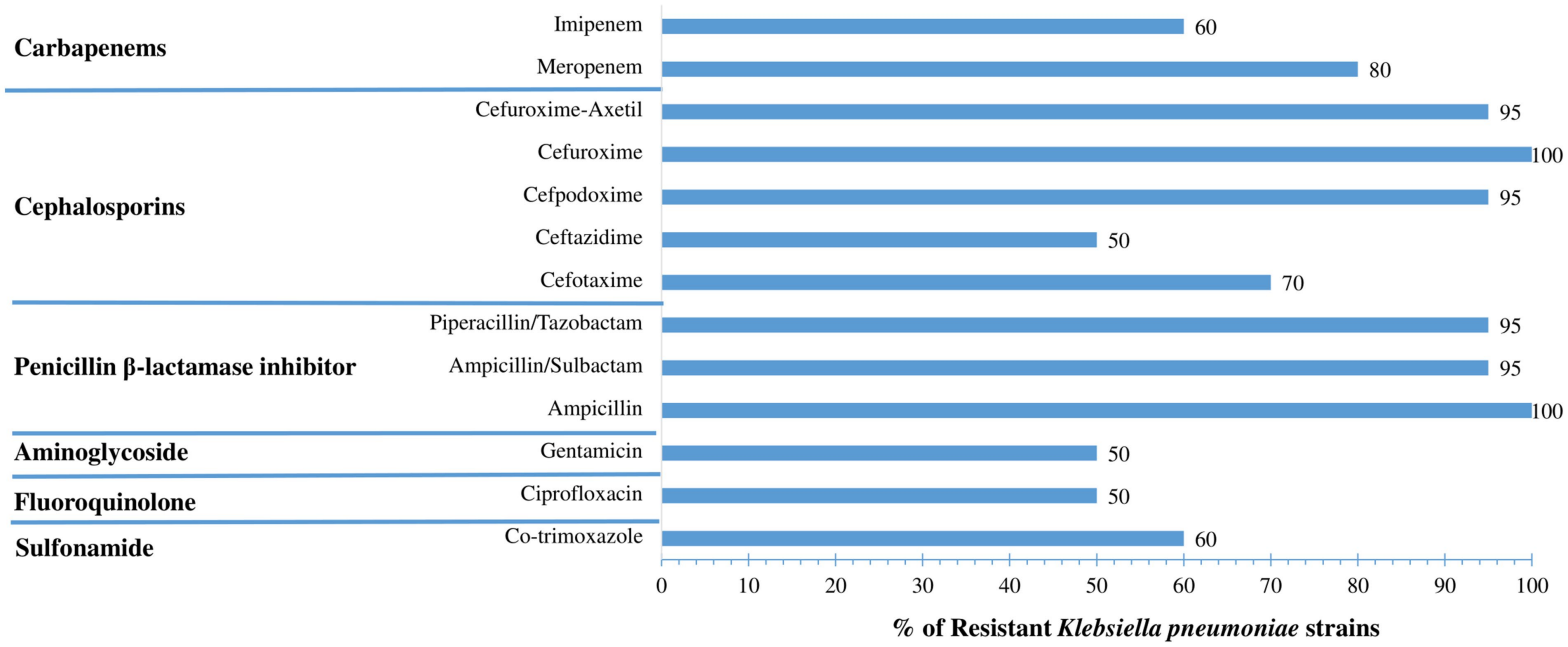
Antimicrobial resistance profile of oral *Klebsiella pneumoniae* isolates. Bar chart represents the percentage of *K. pneumoniae* strains (*n* = 20) resistant to 13 antimicrobial agents across six major antibiotic classes. High levels of resistance were observed to β-lactam agents, with 100% of isolates resistant to ampicillin and cefuroxime, and 95% resistant to cefuroxime-axetil, cefpodoxime, ampicillin/sulbactam, and piperacillin/tazobactam. Resistance to carbapenems was substantial, affecting 80% of isolates for meropenem and 60% for imipenem. A moderate resistance rate (50%) was seen for gentamicin, ciprofloxacin, and ceftazidime, while resistance to cefotaxime and co-trimoxazole was recorded at 70 and 60%, respectively. These findings underscore the multidrug-resistant nature of the isolates and highlight the oral cavity as a potential reservoir for high-risk clones with reduced treatment options.

Antimicrobial susceptibility testing via MICs across 13 antibiotics according to EUCAST recommendations (Supplementary Table 2) showed that nearly all strains of *K. pneumoniae* exhibit resistance to ampicillin, ampicillin/sulbactam, and piperacillin/tazobactam. Notably, high levels of resistance have been reported for carbapenems, with 80% resistance to meropenem and 60% to imipenem. Additionally, high resistance rates were reported for second-generation cephalosporins (95% for cefuroxime and cefuroxime-axetil) and third-generation cephalosporins (95% for cefpodoxime, 70% for cefotaxime, and 50% for ceftazidime), as well as for sulfonamides (60% for trimethoprim/sulfamethoxazole), aminoglycosides (50% for gentamicin), and fluoroquinolones (50% for ciprofloxacin) (Figure 1).

**Figure 2:**
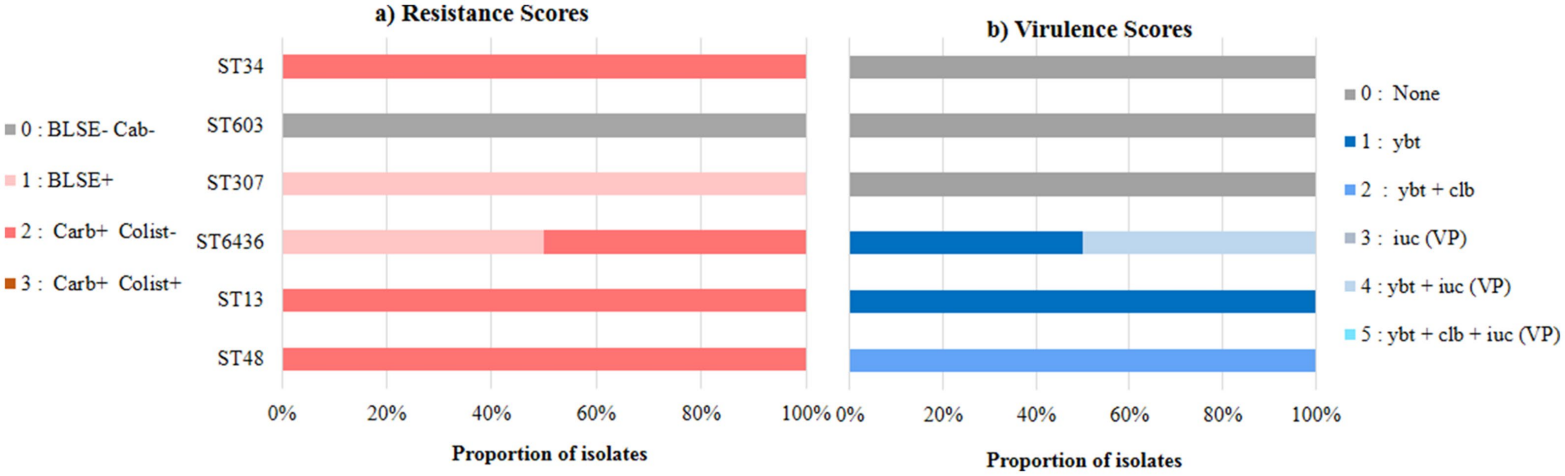
Distribution of **(a)** resistance and **(b)** virulence scores among 20 oral K. pneumoniae isolates. Data shown summarize Kleborate results for WGS K. pneumoniae isolated from oral cavity of hospitalized patients. Lineages were defined on the basis of multi-locus sequence types (STs) reported by Kleborate, and ordered from highest to lowest difference between mean virulence and mean resistance score. Ybt: yersiniabactin, clb: colibactin, iuc: aerobactin, VP: virulence plasmid, ESBL: Extended-Spectrum β-lactamase, Carb: carbapenemase, Colist: colistin resistance determinant.

The WGS performed on the 20 *K. pneumoniae* strains identified 34 acquired antimicrobial resistance (AMR) genes associated with resistance to different classes of antimicrobial agents. Over half (65%) of the *K. pneumoniae* strains were classified as multidrug resistant (MDR), with 50% exhibiting resistance to more than 5 classes of antibiotics (Table 2). Notably, 85% of the isolates showing a high resistance score (score 2) and 50% of these contained more than eight genes per genome. Figure 2 shows the distribution of AMR and virulence scores among the genomes of these *K. pneumoniae* STs, which displays high rates of AMR and/or virulence in ST13 and ST48. Importantly, it also highlights the high rates of AMR and virulence in ST6436 (*n* = 20 AMR genes), which may correspond to AMR-virulence convergence (score 4) within a single strain (K21 isolated from the saliva).

**Table 2:** Clinical and genomic characteristics of *K. pneumoniae* isolates from oral mucosa and saliva samples, including STs, antibiotic resistance.

Strain	Patients	Date	Hospital	Samples^a^	Patients characteristics			ST	β-lactam resistance genes		Aminoglycoside	Fluoroquinolone resistance		Other antibiotics resistance genes	Resistance numbers		Resistance score
					Gender	Age	ATB treatment		Carba	β-lactamase		Mutations QRDR	PMQR		Classes	Genes	
99,029_K01	1	07/05/2023	Akbou	SA	F	42	None	ST13	OXA-48	CTX-M-15, CMY-4, OXA-1, TEM-1D, SHV-1	aac(3)-IIa, strA, strB, aadA2, aac(6′)-Ib-cr		qnrS1	sul1, sul2, dfrA12	7	14	2
99030_K02		07/05/2023	Akbou	OM				ST48	OXA-48	SHV-172				-	1	1	2
99031_K03_SK1	2	07/05/2023	Akbou	SA	F	72	None	ST48	OXA-48	SHV-172				-	1	1	2
99032_K03_SK2		07/05/2023	Akbou	SA				ST48	OXA-48	SHV-172				-	1	1	2
99033_K04_SK1	3	07/05/2023	Akbou	OM	F	89	None	ST48	OXA-48	SHV-172				-	1	1	2
99034_K04_SK2		07/05/2023	Akbou	OM				ST48	OXA-48	SHV-172				-	1	1	2
99035_K05	4	07/05/2023	Akbou	OM	F	67	None	ST13	OXA-48	CTX-M-15, OXA-1, SHV-1	aac(3)-IIa, strA, strB, aadA2, aac(6′)-Ib-cr		qnrS1	sul1, sul2, dfrA12	7	12	2
99036_K06		07/05/2023	Akbou	SA				ST13	OXA-48	CTX-M-15, OXA-1, SHV-1	aac(3)-IIa, strA, strB, aadA2, aac(6′)-Ib-cr		qnrS1	sul1, sul2, dfrA12	7	12	2
99037_K07_SK1	5	07/05/2023	Akbou	OM	F	32	None	ST13	OXA-48	CTX-M-15, CMY-4, CMY-4, CMY-4, OXA-1, TEM-1D SHV-1	aac(3)-IIa, strA, strB, aadA2, aac(6′)-Ib-cr		qnrS1	sul1, sul2, dfr A12	7	16	2
99038_K07_SK2		07/05/2023	Akbou	OM				ST13	OXA-48	CTX-M-15, OXA-1, TEM-1D, SHV-1	aac(3)-IIa, strA, strB, aadA2, aac(6′)-Ib-cr		qnrS1	sul1, sul2, dfrA12	7	13	2
99039_K08		07/05/2023	Akbou	SA				ST13	OXA-48	CTX-M-15, CMY-4, OXA-1, TEM-1D, SHV-1	aac(3)-IIa, strA, strB, aadA2, aac(6′)-Ib-cr		qnrS1	sul1, sul2, dfrA12	7	14	2
99050_K21	8	12/03/2024	Kherrata	SA	F	58	None	ST6436	NDM-5	CTX-M-15, CTX-M-15, OXA-1, TEM-1D, SHV-11	aac(3)-IIa, armA, strA, strB aac(6′)-Ib-cr, aph(3′)-VI,	GyrA-83 ParC-80I	qnrS1	Mrx, mphA, mphE, msrE, sul1, sul2, sul2, dfrA5	8	20	2
99051_K22_SK1	9	12/03/2024	Kherrata	OM	F	55	None	ST307	-	CTX-M-15, OXA-1, TEM-206, SHV-28	aac(3)-IIa, strA, strBaac(6′)-Ib-cr	GyrA-83I ParC-80I	qnrB1	sul2, tet(A), dfrA14	7	11	1
99057_K27	13	05/04/2024	Kherrata	OM	M	29	None	ST6436	-	CTX-M-15, OXA-1, TEM-1D, SHV-11	aac(3)-IIa, strA, strBaac(6′)-Ib-cr	GyrA-83I ParC-80I		sul2	5	8	1
99065_K35	15	05/04/2024	Akbou	OM	F	48	None	ST606-1LV	-	SHV-1					0	0	0
99066_K36	16	05/04/2024	Akbou	OM	F	68	None	ST34	OXA-48	SHV-26					1	1	2
99067_K37	17	05/04/2024	Akbou	OM	M	45	None	ST13	OXA-48	CTX-M-15, OXA-1, TEM-1D, SHV-1	aac(3)-IIa, strA, strB, aadA2, aac(6′)-Ib-cr			sul1, sul2, dfrA12	6	12	2
99070_K39_SK2	18	05/04/2024	Akbou	OM	M	49	None	ST48	OXA-48	SHV-172					1	1	2
99071_K40_SK1	19	05/04/2024	Akbou	OM	F	44	None	ST48	OXA-48	SHV-172					1	1	2
99072_K40_SK2		05/04/2024	Akbou	OM				ST48	OXA-48	SHV-172					1	1	2

a OM, oral mucosa; SA, saliva; Carba, carbapenemase; F, female; M, male.

### *In silico* detection of acquired AMR genes in *K. pneumoniae* strains

The genomic analysis revealed that 17 *K. pneumoniae* strains harbored carbapenemase genes, with 16 strains (85%) positive for *bla*OXA-48 and one strain positive for *bla*NDM*-5*. It was observed that all bacterial species isolated from saliva were positive for carbapenemase production (*bla*OXA-48 = 5 and *bla*NDM-5 = 1), while 64.7% (*n* = 11) of the resistant strains were isolated from oral mucosa swabs (Table 2). Importantly, while carbapenemase genes were clearly associated with an elevation in meropenem MIC, there was variation depending on enzyme type (Supplementary Table 2). NDM-5 was associated with an MIC above the clinical cut-off for resistance (>16 mg/mL); however, OXA-48 enzymes identified were associated with MICs ranging from 4 to >16 mg/mL. Remarkably, although the K36 strain harbors the *bla*OXA*-48* gene, it exhibits MICs of 2 mg/L for both meropenem and imipenem, remaining phenotypically susceptible to these carbapenems. This represents a genotype-positive but phenotype-negative profile. In this study, CRKp classification was primarily based on phenotypic resistance; however, isolates harboring carbapenemase genes were also reported because of their epidemiological relevance.

Among the ESBL genes, 10 strains of *K. pneumoniae* harbored *bla*CTX-M-15, whereas seven strains were associated with *bla*OXA-48. Notably, the strain (K21) that harbored *bla*NDM-5 also contained two determinants of *bla*CTX-M-15 gene. The isolate also harbored broad-spectrum *β*-lactamase (*bla*OXA-1, *bla*TEM-1D, *bla*TEM-206) and AmpC β-lactamase (*bla*CMY-4) encoding genes that are responsible for conferring resistance to β-lactam antibiotics through antibiotic inactivation mechanisms.

Resistance to aminoglycosides was mediated by *aac(3)-IIa* (50%), *aac(6′)-Ib-cr* (50%), *strA* (50%), *strB* (50%), *aadA2* (35%), and *aph(3′)-VI* (5%). Notably, the 16S rRNA methyltransferase (*armA*) gene was also detected in the NDM-5 *K. pneumoniae* K21 strain. Among these genes, the bi-functional gene *aac(6)-Ib-cr* was identified in 10 strains and significantly associated with fluoroquinolone (ciprofloxacin) and aminoglycoside (gentamicin) resistance phenotypes.

In this study, the overall prevalence of PMQR genes in quinolone-resistant bacteria was 50%. The highest frequencies of PMQR genes were related to *aac(6′)-Ib-cr* and *qnrS1* genes, with 50 and 35%, respectively. Additionally, we detected the qnr*B1* gene in one isolate. Screening for chromosomal mutations in the quinolone resistance regions (QRDRs) revealed simultaneous substitutions at codon positions S80I in the ParC gene and S83I in the *GyrA* gene in three isolates. In addition, we observed the co-existence of PMQR genes *aac(6′)-Ib-cr* and *qnrS1* in seven strains, and *aac(6′)-Ib-cr* and *qnrB1* in one strain. Furthermore, in two strains, PMQR genes were associated with QRDR mutations in *gyrA* and *parC*.

Other antimicrobial resistance genes conferring resistance to tetracycline (*tet*A), macrolide (*mrx*, *mph*A, *mph*E, *msr*E), sulfonamide (*sul*1, *sul*2) and trimethoprim (*dfr*A12, *dfr*A5, *dfr*A14) were reported.

### Genomic diversity and clonal relatedness

Whole-genome sequencing of the 20 *K. pneumoniae* isolates revealed genome sizes ranging from 5.45 to 6.02 Mb and a high level of genetic diversity. The genome sequences are available under the BioProject number PRJNA1327787 on the NCBI database. These isolates comprised six distinct sequence types (STs), including internationally recognized clones such as ST13, ST307, and ST48. Some STs were represented by multiple isolates (ST48, *n* = 8; ST13, *n* = 7; ST6436, *n* = 2), whereas others were unique (ST307 and ST34). Most *blaOXA-48*-carrying isolates (*n* = 16) clustered within ST13, ST48, and ST34, while the *blaNDM-5*-positive strain belonged to ST6436. Interestingly, isolates from the same patient included both ST13 (from saliva) and ST48 (from mucosal swabs), suggesting co-colonization by distinct lineages (Table 2).

**Figure 3:**
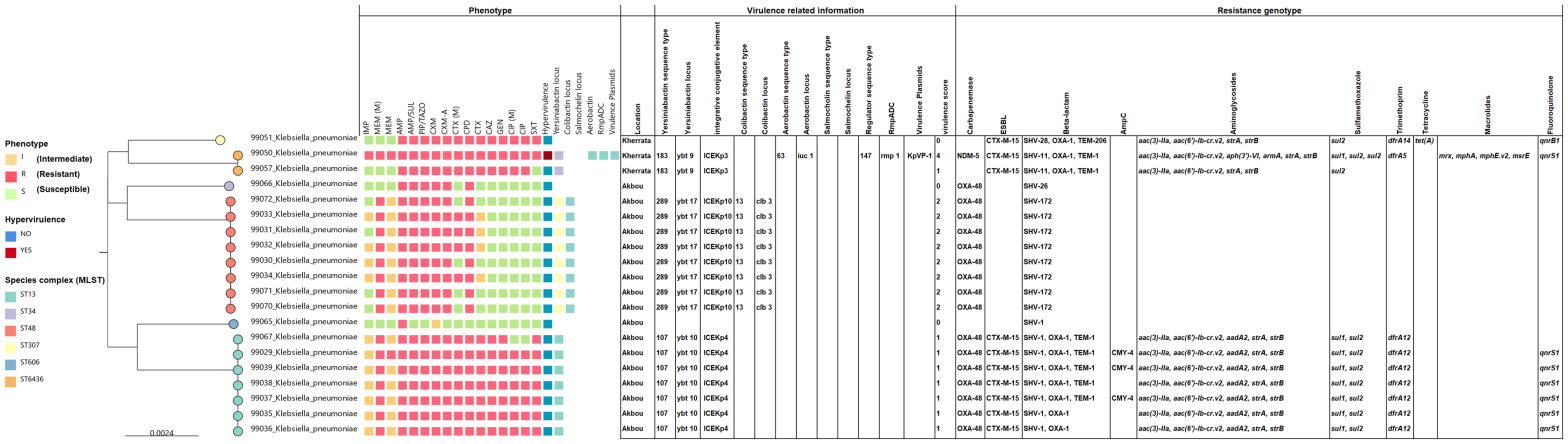
Single-nucleotide polymorphism (SNP)-based phylogenetic tree of *K. pneumoniae* isolates, annotated with resistance and virulence determinants. The phylogeny was constructed using core-genome SNP alignment to infer evolutionary relationships among isolates recovered from oral samples of hospitalized patients. Circles are color-coded according to clonal complexes (ST13, ST34, ST48, ST307, ST606, and ST6436). Nodes are annotated with the antimicrobial resistance phenotype (S, susceptible; I, intermediate; R, resistant), and virulence potential is indicated by color shading (red: hypervirulent; blue: non-virulent). The tree reveals distinct clustering of isolates according to sequence type, reflecting local genetic diversification and horizontal gene transfer events shaping the population structure.

Phylogenetic reconstruction based on core genome SNPs revealed six well-defined clusters corresponding to these STs, confirming a polyclonal population structure (Figure 3). The largest and most heterogeneous clusters, ST13 and ST48, indicate long-term diversification and potential community spread. In contrast, ST307 and ST606 appeared as single-branch clusters, suggesting limited local dissemination, while the two ST6436 isolates, though genetically related, displayed distinct resistance and virulence profiles, illustrating the strong genomic plasticity of *K. pneumoniae*.

The clustering pattern also showed a geographical association: Akbou isolates were distributed across multiple clusters, whereas Kherrata isolates were restricted to two closely related groups, reflecting distinct local lineages influenced by different selective pressures. The co-occurrence of extended-spectrum *β*-lactamases, carbapenemases, and additional resistance genes to aminoglycosides, sulfonamides, trimethoprim, and fluoroquinolones highlights the complexity of multidrug resistance in these environments. Moreover, phylogenetic grouping was associated with the presence of specific integrative and conjugative elements (ICEKp3, ICEKp4, and ICEKp10). The coexistence of *rmp* genes and siderophore systems within the ST6436 lineage suggests an increased potential for hypervirulence, particularly among the Akbou isolates.

### KL and O loci

Among the carbapenem-non-susceptible strains, the following K loci were identified: KL57 (*n* = 7), KL124 (*n* = 8), KL27 (*n* = 1), and KL117 (*n* = 1). Additionally, we found that *K. pneumoniae* K21, isolated from the saliva of a 58-year-old woman and harboring the capsular type K27, was associated with a higher number of virulence and resistance genes (Table 3).

**Table 3:** Virulence genes identified in the oral cavity of the *K. pneumonia.*

Strains	Samples^a^	ST	Virulence profile				Virulence score^b^	K type	K locus	WZI	O locus	O types
			Yersiniabactin	Colibactin	Aerobactin	RmpADC						
99029_K01	SA	ST13	ybt 10; ICEKp4	–	–	–	1	K57	KL57	–	O1/O2v2	O1ab
99030_K02	OM	ST48	ybt 17; ICEKp10	clb 3	–	–	2	Unknown (KL124)	KL124	wzi167	O1/O2v1	O1ab
99031_K03_SK1	SA	ST48	ybt 17; ICEKp10	clb 3	–	–	2	Unknown (KL124)	KL124	wzi167	O1/O2v1	O1ab
99032_K03_SK2	SA	ST48	ybt 17; ICEKp10	clb 3	–	–	2	Capsule null	KL124	wzi167	O1/O2v1	O1ab
99033_K04_SK1	OM	ST48	ybt 17; ICEKp10	clb 3	–	–	2	Unknown (KL124)	KL124	wzi167	O1/O2v1	O1ab
99034_K04_SK2	OM	ST48	ybt 17; ICEKp10	clb 3	–	–	2	Capsule null	KL124	wzi167	O1/O2v1	O1ab
99035_K05	OM	ST13	ybt 10; ICEKp4	–	–	–	1	K57	KL57	–	O1/O2v2	O1ab
99036_K06	SA	ST13	ybt 10; ICEKp4	–	–	–	1	K57	KL57	–	O1/O2v2	O1ab
99037_K07_SK1	OM	ST13	ybt 10; ICEKp4	–	–	–	1	K57	KL57	–	O1/O2v2	O1ab
99038_K07_SK2	OM	ST13	ybt 10; ICEKp4	–	–	–	1	K57	KL57	–	O1/O2v2	O1ab
99039_K08	SA	ST13	ybt 10; ICEKp4	–	–	–	1	K57	KL57	–	O1/O2v2	O1ab
99050_K21	SA	ST6436	ybt 9; ICEKp3	–	iuc 1	rmp 1 KpVP-1	4	K27	KL27	wzi187	O4	O4
99051_K22_SK1	OM	ST307	–	–	–	–	0	Unknown (KL102)	KL102	wzi173	O1/O2v2	O2afg
99057_K27	OM	ST6436	ybt 9; ICEKp3	–	–	–	1	K27	KL27	wzi187	O4	O4
99065_K35	OM	ST606-1LV	–	–	–	–	0	K36	KL36	–	O4	O4
99066_K36	OM	ST34	–	–	–	–	0	Unknown (KL117)	KL117	–	O1/O2v2	O2afg
99067_K37	OM	ST13	ybt 10; ICEKp4	–	–	–	1	K57	KL57	–	O1/O2v2	O1ab
99070_K39_SK2	OM	ST48	ybt 17; ICEKp10	clb 3	–	–	2	Unknown (KL124)	KL124	wzi167	O1/O2v1	O1ab
99071_K40_SK1	OM	ST48	ybt 17; ICEKp10	clb 3	–	–	2	Unknown (KL124)	KL124	wzi167	O1/O2v1	O1ab
99072_K40_SK2	OM	ST48	ybt 17; ICEKp10	clb 3	–	–	2	Unknown (KL124)	KL124	wzi167	O1/O2v1	O1ab

a OM, oral mucosa; SA, saliva.

b Kleborate Virulence Scores (see also: https://github.com/klebgenomics/Kleborate).

K-types were defined according to the wzi alleles. In this study, we observed that the *wzi167* sequence is correlated with an unknown capsular type and non-capsular serotypes, represented by ST48. Other *wzi* allelic types were detected (*wzi187*, *wzi173*) in other sequence types. These results suggest that *wzi* sequencing can distinguish ST48 strains. All seven ST13 strains in our study exhibited capsule type K57, which is known to be associated with liver abscesses and other community-acquired invasive infections, as well as multidrug resistance, while all eight ST48 strains possessed the *wzi*167 (KL124) gene.

We identified three-locus variants O1/O2v2, O1/O2v1, and O4 in this study. The O1 type was predominant, accounting for 15 out of 20 (75%) of the *bla*OXA-48-producing isolates and was associated with either O1/O2v1 or O1/O2v2. However, the O4 antigen was observed across strains carrying *bla*NDM-5, *bla*OXA-48, as well as in sensitive strains. All ST13 strains were KL57/O2v2, whereas all ST148 isolates were KL57/O2v1. We also reported the presence of the O2afg serotype in ST307 harboring *bla*CTX-M-15 and ST34 harboring *bla*OXA-48.

### Virulence detection

Virulence genes encoding yersiniabactin (*ybt*) were the most frequently detected, present in 85% (17/20) of the isolates, followed by colibactin (*clb*) at 40% (8/20). In contrast, aerobactin (*iuc*), salmochelin (*iro*), and hypermucoidy genes (*rmp*ADC) were not found in any of the OXA-48-positive *K. pneumoniae* strains (Table 3).

The hvKp strain (K21) exhibited a high virulence score (4) due to the presence of hypermucoviscosity genes (*rmp1*), yersiniabactin (*ybt9*), and aerobactin (*iuc1*), which together define hypervirulence. This strain also showed a high resistance score (2), carrying 20 resistance genes conferring resistance to eight different antibiotic classes (Table 2, Figure 2). In this study, we refer to the *K. pneumoniae* K21 isolate (ST6436) as a convergent strain, as described by Kochan et al. (2023), because it is resistant to carbapenems and third-generation cephalosporins and harbors hvKP virulence genes. Additionally, we identified three integrative conjugative elements (ICEKp3, ICEKp4, CEKp10), harboring yersiniabactin *ybt*9, *ybt*10, and *ybt*17 genes, respectively. The ICEKp10 harbored both colibactin (*clb*3) virulence factor and yersiniabactin *ybt*17, whereas the ICEKp3 harbored yersiniabactin (*ybt*9), aerobactin (*iuc*1) and *rmp1* genes.

The acquired *rmp*ADC genes are typically mobilized by the large canonical *K. pneumoniae* virulence plasmid (KpVP), while the yersiniabactin gene is mobilized via chromosomal integrative conjugative elements (ICEKp), with variants such as ICEKp10 (*n* = 8), ICEKp4 (*n* = 7), and ICEKp3 (*n* = 2). Notably, the CR-hvKp NDM-5-producing K21 strain has mobilized both integrative conjugative elements (ICEKp3) and the *K. pneumoniae* canonical virulence plasmid (KpVP1) together within its genome (Table 3).

## Discussion

*Klebsiella pneumoniae* is continuously evolving by acquiring resistance, virulence, and other traits that enable it to survive under antimicrobials and immune defenses. This study represents the first genomic characterization of multidrug-resistant (MDR) *K. pneumoniae* isolates from the oral cavity of hospitalized patients in Algeria. We assessed their genetic diversity, resistome, and virulome to evaluate the potential threat posed by international high-risk clones, particularly those combining resistance and hypervirulence.

We reported in this study oral colonization by carbapenem-resistant *K. pneumoniae* in 16 out of 180 hospitalized patients (8.88%) in a medical ward, with ages ranging from 18 to 89 years. Recent investigations have shown that Gram-negative bacteria resistant to third-generation cephalosporins or carbapenems such as *Acinetobacter*, *Pseudomonas*, and *Enterobacterales* can be isolated from the oral cavities of various populations. These include elderly individuals in long-term care facilities (Haruta et al., 2023; Kajihara et al., 2023; Nishihama et al., 2025), healthy adolescents (Kawayanagi et al., 2024), and healthy subjects with no prior antibiotic exposure, such as neonates and breastfed infants (Milanović et al., 2020). Understanding the oral resistome provides valuable insights into the most prevalent antimicrobial resistance genes (ARGs), their mobilization potential, and transmission risk via oral bacteria (Sukumar et al., 2024). The oral cavity is particularly important for investigation due to its anatomical and physiological features, which favor microbial proliferation and transmission through oropharyngeal secretions and saliva droplets when talking, coughing, sneezing, breathing, or kissing (De Paula Campêlo et al., 2022; Sukumar et al., 2024). Effectively, carbapenemase-producing *K. pneumoniae* were detected in patients, their environment, and their oral and rectal samples, highlighting potential hospital-associated environmental contamination (Wei et al., 2021). In this study, the majority of *K. pneumoniae* strains (14/20) were isolated from oral mucosa, while six were isolated from saliva samples, with six patients harboring multiple distinct resistant strains in their oral cavity (Supplementary Table 1). In healthy individuals, Gram-negative bacilli sporadically colonize the oral cavity and are not considered natural components of the oral microbiota. However, colonization by *Enterobacteriaceae* is a dynamic process influenced by hospitalization, compromised immunological response, poor oral hygiene, reduced salivary flow, and impaired mastication (Leão-Vasconcelos et al., 2015). Their incidence is variable and may increase under specific conditions such as diabetes (Li et al., 2000; Ashreen et al., 2020), advanced age (Li et al., 2000; Cruz et al., 2022), cancer (Santibañez-Bedolla et al., 2023), and HIV (Arirachakaran et al., 2019), confirming that their presence can be a marker of clinical severity. In the present study, despite limited clinical information, diabetes mellitus was the most frequently documented comorbidity among patients colonized with carbapenem-resistant *K. pneumoniae*, consistent with previous studies showing that diabetes significantly increases the risk of colonization and infection by CRKp (Pattolath et al., 2024; Wang et al., 2025).

Gram-negative bacteria have been associated with several oral diseases, including periodontitis, caries, and gingivitis, as well as systemic conditions of clinical relevance (Zaatout, 2021; Bridon, 2024). Inappropriate antibiotic use for these diseases may favor the selection and persistence of resistant bacteria in the oral cavity, including ESBL and carbapenemase-producing strains (Katkowska et al., 2023; Bridon, 2024). Cruz et al. (2022) reported that pathogenic microorganisms can colonize the oral cavity within 48 h of hospital admission, with host-related factors such as age, frailty, and comorbidities playing a greater role than hospitalization duration (Cruz et al., 2022). In contrast, De Paula Campêlo et al. (2022) reported a rapid shift in the oral microbiota of critically ill adult patients, characterized by the predominance of multidrug-resistant hospital-associated Gram-negative and/or Gram-positive bacteria. These studies suggest that these microorganisms can regularly colonize the oral cavity, but it is not well established whether this presence is transient (Cruz et al., 2022). These findings suggest that multidrug-resistant bacteria may be present in the oral cavity of both hospitalized patients and the general population. Given the potential for both community and hospital acquisition, the source of the *K. pneumoniae* isolates identified in this study cannot be definitively determined. Nevertheless, one possible route of entry for these bacteria is through water and food, as the incidence of coliforms and other bacteria in these sources may contribute to oral colonization (Leão-vasconcelos et al., 2015). Supporting this hypothesis, *K. pneumoniae* producing OXA-48 and ESBL have been detected in fresh vegetables in Algeria, in the same city as our hospitalized patients, highlighting the widespread occurrence of multidrug-resistant Gram-negative bacteria in agri-food chains, particularly in vegetables and fruits consumed raw (Touati et al., 2017). In addition, long-term dietary habits may contribute to the presence of antibiotic resistance genes in the mouths of healthy adults, reflecting the widespread use of antibiotics in humans, animals, and agriculture (Milanović et al., 2020).

Notably, 65% of the isolates were multidrug-resistant (MDR), with 85% reaching a resistance score of 2, exhibiting resistance across up to five antibiotic classes and highlighting the oral cavity as a hidden reservoir of MDR *K. pneumoniae* in hospitalized patients (Table 2, Figure 1). Approximately 80% of isolates were resistant to at least one of the tested carbapenems, with the majority carrying the *bla*OXA-48 gene (94.11%), while only one isolate (K21) harbored *bla*NDM-5. Half of the isolates co-produced extended-spectrum *β*-lactamase (ESBL), primarily *bla*CTX-M-15. Remarkably, the K21 strain was classified as extensively drug-resistant (XDR), exhibiting resistance to more than five antibiotic classes and harboring 20 resistance genes (Table 2), underscoring the complexity of resistance mechanisms in oral *K. pneumoniae*. The emergence of carbapenemase-producing bacteria has become a major public health concern in recent years. Carbapenemases are most often encoded on mobile genetic elements such as plasmids that can be easily transferred across species, including among *Enterobacteriaceae*, through horizontal gene transfer (Xie et al., 2025).

The high prevalence of carbapenemase-producing isolates observed in this study is consistent with the alarming levels of antimicrobial resistance (AMR) reported by the Algerian Antibiotic Resistance Surveillance Network.^1^ Increasing rates of carbapenem and ESBL resistance, particularly among *K. pneumoniae*, confirm that antibiotic resistance in Algeria has reached critically concerning levels. Several studies have shown that the OXA-48 enzyme is the predominant carbapenemase among hospitalized patients, confirming its endemicity in Algeria (Abderrahim et al., 2017; Bourafa et al., 2018; Zemmour et al., 2021; Abderrahim et al., 2022). This enzyme is also widely disseminated across the Mediterranean region and African countries (Mathlouthi et al., 2017; Peyclit et al., 2018; Touati and Mairi, 2020; Chelaru et al., 2024). In parallel, the NDM-5 gene, first reported in multidrug-resistant *E. coli* from Algeria in 2014 (Sassi et al., 2014), has since been detected in human, animal, and environmental sources within the country, including pets (Yousfi et al., 2015), clinical isolates (Khaldi et al., 2022; Nabti et al., 2022), and migratory birds (Loucif et al., 2022b), highlighting its extensive dissemination across One Health compartments. The detection of NDM-5 in the oral cavity of a hospitalized patient in our study further underscores the widespread circulation of this carbapenemase in Algeria.

Phylogenetic analysis of the oral *K. pneumoniae* isolates revealed substantial clonal and phenotypic diversity, particularly among samples from Akbou hospital (Figure 3), suggesting local adaptation driven by antibiotic pressure and environmental factors. This heterogeneity between isolates from different hospitals likely reflects distinct selective pressures and horizontal gene transfer dynamics, contributing to the emergence of multiple clonal lineages within the same species. Whole-genome sequencing revealed genome sizes ranging from 5.45 to 6.02 Mb, consistent with previous reports describing the large, plastic genomes of *Klebsiella* species enriched in mobile genetic elements (Wyres and Holt, 2018; Rocha et al., 2022). *The K. pneumoniae* genome contains approximately 5,500 genes, of which about 3,500 constitute the accessory genome, encoding numerous resistance and virulence factors (Wyres and Holt, 2018; Rocha et al., 2022). Liu et al. (2024) reported that *Klebsiella* species are among the few oral and nasal bacteria characterized by remarkable metabolic versatility that enables adaptation to multiple environments. This genomic flexibility underpins the species’ ability to acquire resistance and virulence genes, supporting its opportunistic and adaptive lifestyle. In line with this, our assemblies identified genes associated with siderophore production, antimicrobial resistance, and diverse virulence functions that collectively enhance bacterial persistence under adverse conditions. This genomic plasticity was further illustrated by the detection of resistance genes with variable phenotypic expression. The detection of the *blaOXA-48* gene in *K. pneumoniae* K36, despite its low MIC (2 mg/L) to both meropenem and imipenem, highlights a notable discrepancy between genotype and phenotype. Notably, this finding underlines an important limitation of phenotype-based definitions of carbapenem-resistant *K. pneumoniae* (CRKp), as it harbors the carbapenemase gene *bla*OXA-48 while remaining phenotypically susceptible to imipenem and meropenem. This highlights that phenotypic testing, although rapid and clinically indispensable, may fail to identify genotypic resistance determinants that are highly relevant for therapeutic decision-making and epidemiological surveillance. Similar observations have been reported in previous studies, where OXA-48 producers frequently exhibit low-level carbapenem resistance, often falling below EUCAST (≤2 mg/L) and CLSI (≤1 mg/L) clinical breakpoints (Boyd et al., 2022; Meekes et al., 2025). This inconsistency may result from low-level expression of the gene, potentially influenced by its promoter context, plasmid copy number, or the absence of permeability defects (porin loss) that are often required to achieve full carbapenem resistance. Such silent carriers pose a risk, as they can evade routine susceptibility testing while still contributing to the spread of resistance genes. Alternatively, the strain may carry a non-functional or weakly active *bla*OXA-48 allele, further explaining the low phenotypic resistance. The contrasting resistance and virulence profiles observed among the two ST6436 (K21 and K27) isolates may also reflect the population heterogeneity within *K. pneumoniae in the oral cavity*. This heterogeneity, arising from genetic variation and phenotypic plasticity, represents an adaptive “bet-hedging” strategy that enhances bacterial survival under fluctuating environmental conditions, including antibiotic exposure (Cho et al., 2025). The identification of a convergent ST6436 (K21) strain in the oral cavity, carrying both resistance and virulence determinants, raises important clinical concerns. Oral bacteria can enter the bloodstream during routine activities or dental procedures such as tooth brushing, chewing, or periodontal surgery, potentially leading to systemic infections such as bacteremia or pneumonia (Gendron et al., 2000; Kawayanagi et al., 2024). Moreover, the oral cavity may facilitate horizontal gene transfer to gut microbiota, promoting the dissemination of antimicrobial resistance and virulence traits within and beyond the host, particularly in hospitalized or immunocompromised patients (Kawayanagi et al., 2024).

Resistance and virulence in *K. pneumoniae* are frequently linked to specific clonal lineages (Aghamohammad et al., 2023). While global attention has mainly focused on well-known high-risk clones, other multidrug-resistant lineages capable of disseminating carbapenem resistance remain underexplored. In this study, whole-genome sequencing revealed the ongoing spread of two OXA-48-producing high-risk clones, ST13 and ST48, which harbor a substantial repertoire of resistance and virulence determinants. ST13 *K. pneumoniae* is an internationally disseminated high-risk clone associated with the spread of several ESBL and carbapenemase genes, including *bla*OXA-48 (Fursova et al., 2020; Österblad et al., 2012; Wrenn et al., 2014), *bla*KPC (Elias et al., 2023; Mendes et al., 2023), and *bla*CTX-M-15 (Marcade et al., 2013) across Europe. In Algeria, ST13 represents the most prevalent *K. pneumoniae* strains producing the OXA-48 lineage, recovered from diverse environmental and clinical sources (Touati and Mairi, 2020), including human fecal carriage, river water, farm animal feces, wild fish intestines, milk, wild bird feces, and various food products. This lineage was reported from both Bejaia (the same city as our study) and Tizi Ouzou province indicating a potential regional dissemination of this clone (Mairi et al., 2019; Touati and Mairi, 2020; Zemmour et al., 2021). Moreover, ST13 has been identified in hospital environments in Constantine province, carrying *CTX-M-15*, *aac(6’)Ib-cr*, and *aadA2* genes conferring resistance to *β*-lactams, quinolones, and aminoglycosides (Zenati et al., 2017).

ST 48 was the founder ST of the clonal complex CC48 (Naha et al., 2021). In Algeria, Chaalal et al. (2021) showed the clonal expansion of *K. pneumoniae* isolates with the dissemination of ST48 among OXA-48-producing strains in chicken meat from three poultry farms in Western Algeria, indicating that these *Klebsiella* isolates could be easily transmitted to humans from food animals and their products. ST48 was also previously detected in an urban wastewater treatment plant in Algeria (Chaalal et al., 2021). This ST was also reported in the clonal dissemination of *K. pneumoniae* producing KPC-2 among hospitalized patients in China (Gu et al., 2019) and from *K. pneumoniae* isolates harboring the *bla*OXA-181 gene in neonates in India (Naha et al., 2021).

*K. pneumoniae* ST307 is a globally disseminated high-risk clone commonly associated with ESBL production (*bla*CTX-M-15) and a strong ability to acquire carbapenemase genes such as *bla*OXA-48, *bla*KPC, and *blaN*DM (Núñez-Samudio et al., 2022; Aghamohammad et al., 2023). In the present study, the ST307 isolate harbored a similar resistance profile and showed high genetic relatedness to strains previously reported in tertiary healthcare settings in Uganda (Byarugaba et al., 2023). However, in contrast to the Ugandan isolates, our strain lacked virulence-associated genes, suggesting local genomic variation and possible early-stage adaptation of this lineage to the local environment. Comparable intra-lineage diversity has been described in other globally circulating clones, including ST307, ST147, and ST15, underscoring the remarkable genetic plasticity of *K. pneumoniae* under diverse ecological and selective pressures (Wyres and Holt, 2018; Núñez-Samudio et al., 2022). Iron acquisition through siderophore production represents a key adaptive and virulence-associated trait in *Klebsiella pneumoniae*. Several siderophore systems, including yersiniabactin, aerobactin, and the Klebsiella ferric uptake system (kfu), have been described in clinical isolates, with highly heterogeneous distribution across sequence types (Di Mento et al., 2022). In the present study, the predominance of yersiniabactin (17/20), followed by colibactin (8/20), among oral isolates highlights their role in adaptation to the oral environment and suggests a contribution to enhanced bacterial fitness and persistence rather than overt invasiveness. We also confirmed the presence of the same iron uptake system genes in ST13 (*ybt*10), ST48 (*ybt*17 and *clb*3), as well as the absence of iron chelation mechanism genes in ST34, ST606-1LV, and ST307 strains. Interestingly, intra-lineage variability was observed within ST6436. *K. pneumoniae* K21 displayed yersiniabactin (*ybt*9) and aerobactin (*iuc*1) and hypermucoidy (*rmp*1) genes, whereas *K. pneumoniae* K27 harbored only yersiniabactin (*ybt*9). These findings illustrate the virulome divergence among *K. pneumoniae* strains from the oral cavity of hospitalized patients in Bejaia province, Algeria. Furthermore, multidrug-resistant lineages ST13 (OXA-48; CTX-M-15), ST48(OXA-48), and ST6436 (NDM-5; CTX-M-15) were associated with *ybt*10/ICEKp4, *ybt*17/ICEKp10, and *ybt* 9/ICEKp3, respectively. This heterogeneity confirms that siderophore acquisition is lineage-specific and primarily shaped by local ecological and selective pressures, rather than being universally conserved among multidrug-resistant clones, as previously reported by Di Mento et al. (2022).

We also report for the first time the predominance of integrative conjugative elements ICEKp (80%) variants carrying the *bla*OXA-48 gene associated with yersiniabactin variants in our strains. Integrative conjugative elements carrying antimicrobial resistance and/or virulence determinants are common in the opportunistic pathogen *K. pneumoniae*, which is a leading cause of highly drug-resistant infections in hospitals (Lam et al., 2018). The widespread detection of the yersiniabactin gene and its associated ICEKp among clinical *K. pneumoniae* producing OXA-48 has been reported in Spain and the Netherlands (Jati et al., 2023) but never in Algeria. Here, we also report the co-existence of ICEKp3 and KpVP-1 in the convergent *K. pneumoniae* K21 producing NDM-5 and harboring the iron acquisition systems yersiniabactin (*ybt*9), aerobactin (*iuc*1), and *rmp*A (*rmp*1).

The capsular types commonly associated with hvKp/CR-hvKp are, in order of frequency, K1, K2, and K57 (García-Cobos et al., 2025). In our study, K57 was associated with ST13 strains harboring yersiniabactin, *bla*OXA-48, and *bla*CTX-M-15 genes and other determinants.

Notably, the capsular type K27 was identified in two ST6436 isolates showing remarkable intra-lineage divergence. One isolate (strain K21) represented a convergent hv-CRKp harboring *bla*NDM-5, *bla*CTX-M-15, yersiniabactin (*ybt9*), aerobactin (*iuc*1), *rmp*1, and wzi, whereas the other isolate (strain K27) carried only *bla*CTX-M-15, yersiniabactin (*ybt9*), and wzi, illustrating divergent virulence profiles within the same clonal lineage sharing the same capsular type. The *wzi* gene, essential for capsular polysaccharide synthesis, was detected in 11 isolates, underscoring its role in antimicrobial resistance and biofilm formation (Alnaqeeb and Gergees, 2024). Additionally, most isolates possessed the lipopolysaccharide serotype O1v1, while O2afg was observed only in two isolates (K22 and K36). Importantly, the O2a and O2afg serotypes are prevalent in most multidrug-resistant *K. pneumoniae* strains and have been shown to induce poor inflammatory immune responses, promoting immune evasion and subsequent survival of CR-Kp strains (Bulati et al., 2021; Di Mento et al., 2022). These observations underscore the complexity of the mechanisms employed by multidrug-resistant *K. pneumoniae* to persist within the host and promote onward transmission among patients. These findings contribute to the growing body of evidence on convergent strains emerging across Africa. In countries such as Egypt, Nigeria, and Ghana, the combination of MDR and hypervirulence has been documented, with ST11-KL47 and ST147-K67 being the most notable examples (García-Cobos et al., 2025). In Algeria specifically, diverse capsular types including K1 (ST23), K2 (ST14 and ST86), K3 (ST13), K31 (ST2108), K6 (ST348), and K151 (ST405) have been identified in ESBL-producing and virulent *K. pneumoniae* from hospitalized patients at Oran hospital (Zemmour et al., 2021), highlighting the capsular diversity within the country. The identification of K57 and K27 types in our study from Bejaia further underscores this regional variability.

In conclusion, this study provides the first genomic characterization of oral carbapenemase-producing *K. pneumoniae* in hospitalized patients in Algeria, revealing widespread dissemination of OXA-48 and NDM-5 among high-risk lineages. The oral cavity serves as a reservoir of highly pathogenic strains with transmission potential via droplets and contamination. The discovery of convergent strains, such as ST6436-K21 harboring NDM-5, iuc1, and rmpA, in the oral cavity supports the hypothesis of hidden reservoirs outside the gut, challenging the paradigm that MDR and hvKp strains are separate or only gut-associated.

Our results demonstrate that greater attention should be given to oral bacteria for antimicrobial resistance prevention, highlighting the importance of maintaining oral health as a preventive measure. This identification is of utmost importance due to the fact that for a long time, MDR-Kp and hvKp have been considered distinct phenotypes associated with different patient populations. Whole-genome sequencing has proven valuable for characterizing *K. pneumoniae*, enabling monitoring of genetic changes, understanding transmission, and tackling the spread of hv*Kp*. Our findings highlight the risk of MDR and hypervirulent convergent strains within Algerian hospitals. While carbapenem resistance detection is routine, virulence gene detection is not, and phenotypic methods such as the string test lack sensitivity. The convergence of hypervirulence and multidrug resistance is no longer confined to the gut or bloodstream but also extends to the oral ecosystem, representing an unrecognized risk for nosocomial and community transmission.

## Data Availability

The datasets presented in this study can be found in online repositories. The names of the repository/repositories and accession number(s) can be found below: https://www.ncbi.nlm.nih.gov/, SAMN51292627 https://www.ncbi.nlm.nih.gov/, SAMN51292628 https://www.ncbi.nlm.nih.gov/, SAMN51292629 https://www.ncbi.nlm.nih.gov/, SAMN51292630 https://www.ncbi.nlm.nih.gov/, SAMN51292631 https://www.ncbi.nlm.nih.gov/, SAMN51292632 https://www.ncbi.nlm.nih.gov/, SAMN51292633 https://www.ncbi.nlm.nih.gov/, SAMN51292634 https://www.ncbi.nlm.nih.gov/, SAMN51292635 https://www.ncbi.nlm.nih.gov/, SAMN51292636 https://www.ncbi.nlm.nih.gov/, SAMN51292637 https://www.ncbi.nlm.nih.gov/, SAMN51292640 https://www.ncbi.nlm.nih.gov/, SAMN51292645 https://www.ncbi.nlm.nih.gov/, SAMN51292647 https://www.ncbi.nlm.nih.gov/, SAMN51292648 https://www.ncbi.nlm.nih.gov/, SAMN51292649 https://www.ncbi.nlm.nih.gov/, SAMN51292650 https://www.ncbi.nlm.nih.gov/, SAMN51292651 https://www.ncbi.nlm.nih.gov/, SAMN51292652 https://www.ncbi.nlm.nih.gov/, SAMN51292641.

## Footnotes

1
www.aarn.pasteur.dz
