# Supplementary material for: The oral cavity as a reservoir for resistance- and hypervirulence-associated genes of Klebsiella pneumoniae in hospitalized patients
Source: Front Microbiol. 2026 Feb 5;17:1751947. doi: 10.3389/fmicb.2026.1751947 (PMC12916564; doi:10.3389/fmicb.2026.1751947)
Supplement: Supplementary file 1 [file Table_1.docx]

**Supplementary data**

**Table 1: Characteristics of participants screened for carriage *Klebsiella pneumoniae***

| **Strains** | **Patients** | **WGS identification** | **Ward** | **Date/Year** | **Hospital** | **Samples** | **Patients characteristics** | | | |
| --- | --- | --- | --- | --- | --- | --- | --- | --- | --- | --- |
| **Codes** |  |  |  |  |  |  | **Gender** | **Age** | **ATB treatment** | **Hospitalization status** |
| 99029_KLEP_K01 | Patient 1 | *K. pneumoniae* | Medical | 07/05/2023 | Akbou | Saliva | Female | 42 | Non | Diabetes mellitus |
| 99030_KLEP_K02 |  | *K. pneumoniae* | Medical | 07/05/2023 | Akbou | Oral mucosa |  |  |  |  |
| 99031_KLEP_K03_SK1 | Patient 2 | *K. pneumoniae* | Medical | 07/05/2023 | Akbou | Saliva | Female | 72 | Non | Scleroderma |
| 99032_KLEP_K03_SK2 |  | *K. pneumoniae* | Medical | 07/05/2023 | Akbou | Saliva |  |  |  |  |
| 99033_KLEP_K04_SK1 | Patient 3 | *K. pneumoniae* | Medical | 07/05/2023 | Akbou | Oral mucosa | Female | 89 | Non | Thyroid disorders |
| 99034_KLEP_K04_SK2 |  | *K. pneumoniae* | Medical | 07/05/2023 | Akbou | Oral mucosa |  |  |  |  |
| 99035_KLEP_K05 | Patient 4 | *K. pneumoniae* | Medical | 07/05/2023 | Akbou | Oral mucosa | Female | 67 | Non | Diabetes mellitus |
| 99036_KLEP_K06 |  | *K. pneumoniae* | Medical | 07/05/2023 | Akbou | Saliva |  |  |  |  |
| 99037_KLEP_K07_SK1 | Patient 5 | *K. pneumoniae* | Medical | 07/05/2023 | Akbou | Oral mucosa | Female | 32 | Non | Diabetes mellitus |
| 99038_KLEP_K07_SK2 |  | *K. pneumoniae* | Medical | 07/05/2023 | Akbou | Oral mucosa |  |  |  |  |
| 99039_KLEP_K08 |  | *K. pneumoniae* | Medical | 07/05/2023 | Akbou | Saliva |  |  |  |  |
| 99050_KLEP_K21 | Patient 8 | *K. pneumoniae* | Medical | 12/03/2024 | Kherrata | Saliva | Female | 58 | Non | Diabetes mellitus |
| 99051_KLEP_K22 | Patient 9 | *K. pneumoniae* | Medical | 12/03/2024 | Kherrata | Oral mucosa | Female | 55 | Non | Hypertension |
| 99057_KLEP_K27 | Patient 13 | *K. pneumoniae* | Medical | 05/04/2024 | Kherrata | Oral mucosa | Male | 29 | Non | Hypertension |
| 99065_KLEP_K35 | Patient 15 | *K. pneumoniae* | Medical | 05/04/2024 | Akbou | Oral mucosa | Female | 48 | Non | Thyroid disorders |
| 99066_KLEP_K36 | Patient 16 | *K. pneumoniae* | Medical | 05/04/2024 | Akbou | Oral mucosa | Female | 68 | Non | Surgery |
| 99067_KLEP_K37 | Patient 17 | *K. pneumoniae* | Medical | 05/04/2024 | Akbou | Oral mucosa | Male | 45 | Non | Respiratory diseases |
| 99070_KLEP_K39 | Patient 18 | *K. pneumoniae* | Medical | 05/04/2024 | Akbou | Oral mucosa | Male | 49 | Non | Thyroid disorders |
| 99071_KLEP_K40_SK1 | Patient 19 | *K. pneumoniae* | Medical | 05/04/2024 | Akbou | Oral mucosa | Female | 44 | Non | Stroke |
| 99072_KLEP_K40_SK2 |  | *K. pneumoniae* | Medical | 05/04/2024 | Akbou | Oral mucosa |  |  |  |  |

**Table 2 : Antimicrobial susceptibility testing of Klebsiella *pneumoniae* by MIC determination**

| **Code strains** | **Species** | **Origine** | **Carba** | **Antibiotic MICs** | | | | | | | | | | | | |
| --- | --- | --- | --- | --- | --- | --- | --- | --- | --- | --- | --- | --- | --- | --- | --- | --- |
|  |  |  |  | **AMP** | **AMP/**  **Sul** | **PIP/**  **TAZ** | **CEFRO** | **CEFRO/**  **AXE** | **CEFPO** | **CTX** | **CAZ** | **IMP** | **MERO** | **GEN** | **CIP** | **TRIM/**  **Sulf** |
| 99029_KLEP_K01 | *K. pneumoniae* | SA | OXA-48 | ≥ 32 | ≥ 32 | ≥ 128 | ≥ 64 | ≥ 64 | ≥ 8 | ≥ 64 | ≥ 64 | 4 | ≥ 16 | ≥ 16 | ≥ 4 | ≥ 320 |
| 99030_KLEP_K02 | *K. pneumoniae* | OM | OXA-48 | ≥ 32 | ≥ 32 | ≥ 128 | 4 | 4 | ≤ 0,25 | 1 | ≤ 0,12 | 4 | 4 | ≤ 1 | ≤ 0,06 | ≤ 20 |
| 99031_KLEP_K03_SK1 | *K. pneumoniae* | SA | OXA-48 | ≥ 32 | ≥ 32 | ≥ 128 | 4 | 4 | ≤ 0,25 | 2 | 0.25 | 4 | 4 | ≤ 1 | ≤ 0,06 | ≤ 20 |
| 99032_KLEP_K03_SK2 | *K. pneumoniae* | SA | OXA-48 | ≥ 32 | ≥ 32 | ≥ 128 | 4 | 4 | ≤ 0,25 | 2 | 0.25 | 2 | 8 | ≤ 1 | ≤ 0,06 | ≤ 20 |
| 99033_KLEP_K04_SK1 | *K. pneumoniae* | OM | OXA-48 | ≥ 32 | ≥ 32 | ≥ 128 | 4 | 4 | ≤ 0,25 | 2 | ≤ 0,12 | 4 | 4 | ≤ 1 | ≤ 0,06 | ≤ 20 |
| 99034_KLEP_K04_SK2 | *K. pneumoniae* | OM | OXA-48 | ≥ 32 | ≥ 32 | ≥ 128 | 4 | 4 | ≤ 0,25 | 2 | 0.25 | 4 | 4 | ≤ 1 | ≤ 0,06 | ≤ 20 |
| 99035_KLEP_K05 | *K. pneumoniae* | OM | OXA-48 | ≥ 32 | ≥ 32 | ≥ 128 | ≥ 64 | ≥ 64 | ≥ 8 | ≥ 64 | 8 | 4 | 4 | ≥ 16 | 1 | ≥ 320 |
| 99036_KLEP_K06 | *K. pneumoniae* | SA | OXA-48 | ≥ 32 | ≥ 32 | ≥ 128 | ≥ 64 | ≥ 64 | ≥ 8 | ≥ 64 | 8 | 4 | 4 | ≥ 16 | 1 | ≥ 320 |
| 99037_KLEP_K07_SK1 | *K. pneumoniae* | OM | OXA-48 | ≥ 32 | ≥ 32 | ≥ 128 | ≥ 64 | ≥ 64 | ≥ 8 | ≥ 64 | ≥ 64 | 4 | 8 | ≥ 16 | ≥ 4 | ≥ 320 |
| 99038_KLEP_K07_SK2 | *K. pneumoniae* | OM | OXA-48 | ≥ 32 | ≥ 32 | ≥ 128 | ≥ 64 | ≥ 64 | ≥ 8 | ≥ 64 | ≥ 64 | 4 | 8 | ≥ 16 | ≥ 4 | ≥ 320 |
| 99039_KLEP_K08 | *K. pneumoniae* | SA | OXA-48 | ≥ 32 | ≥ 32 | ≥ 128 | ≥ 64 | ≥ 64 | ≥ 8 | ≥ 64 | ≥ 64 | 4 | 8 | ≥ 16 | ≥ 4 | ≥ 320 |
| 99050_KLEP_K21 | *K. pneumoniae* | SA | NDM-5 | ≥ 32 | ≥ 32 | ≥ 128 | ≥ 64 | ≥ 64 | ≥ 8 | ≥ 64 | ≥ 64 | 8 | ≥ 16 | ≥ 16 | ≥ 4 | ≥ 320 |
| 99051_KLEP_K22_SK1 | *K. pneumoniae* | OM | OXA-48 | ≥ 32 | ≥ 32 | ≥ 128 | ≥ 64 | ≥ 64 | ≥ 8 | ≥ 64 | 32 | ≤ 0,25 | ≤ 0,25 | ≥ 16 | ≥ 4 | ≥ 320 |
| 99057_KLEP_K27 | *K. pneumoniae* | OM | OXA-48 | ≥ 32 | ≥ 32 | 8 | ≥ 64 | ≥ 64 | ≥ 8 | ≥ 64 | 8 | ≤ 0,25 | ≤ 0,25 | ≤ 16 | ≥ 4 | ≥ 20 |
| 99065_KLEP_K35 | *K. pneumoniae* | OM | OXA-48 | ≥ 32 | 4 | ≤ 4 | ≤ 1 | ≤ 1 | ≤ 0,25 | ≤ 0,25 | ≤ 0,12 | ≤ 0,25 | ≤ 0,25 | ≤ 1 | ≤ 0,06 | ≤ 20 |
| 99066_KLEP_K36 | *K. pneumoniae* | OM | OXA-48 | ≥ 32 | ≥ 32 | ≥ 128 | 4 | 4 | ≤ 0,25 | 1 | 0.25 | 2 | 2 | ≤ 1 | ≤ 0,06 | ≤ 20 |
| 99067_KLEP_K37 | *K. pneumoniae* | OM | OXA-48 | ≥ 32 | ≥ 32 | ≥ 128 | ≥ 64 | ≥ 64 | ≥ 8 | ≥ 64 | 32 | 4 | 8 | ≥ 16 | 0.12 | ≥ 320 |
| 99070_KLEP_K39_SK2 | *K. pneumoniae* | OM | OXA-48 | ≥ 32 | ≥ 32 | ≥ 128 | 4 | 4 | ≤ 0,25 | 1 | 0.25 | 2 | 4 | ≤ 1 | ≤ 0,06 | ≤ 20 |
| 99071_KLEP_K40_SK1 | *K. pneumoniae* | OM | OXA-48 | ≥ 32 | ≥ 32 | ≥ 128 | 4 | 4 | ≤ 0,25 | 1 | ≤ 0,12 | 2 | 4 | ≤ 1 | ≤ 0,06 | ≤ 20 |
| 99072_KLEP_K40_SK2 | *K. pneumoniae* | OM | OXA-48 | ≥ 32 | ≥ 32 | ≥ 128 | 4 | 4 | ≤ 0,25 | 1 | 0.25 | 2 | 4 | ≤ 1 | ≤ 0,06 | ≤ 20 |

**OM : Oral Mucosa, SA: Saliva, Carba : Carbapenemase**
